# Establishment of precise prevention strategies for the occurrence and progression of coronary atherosclerotic heart disease using machine learning

**DOI:** 10.1016/j.heliyon.2024.e35797

**Published:** 2024-08-03

**Authors:** Qingfeng Wu, Huiyi Wei, Cong Lu, Xiaoxian Chi, Rongfang Li, Qingbin Zhao

**Affiliations:** a Department of Geratology, The First Affiliated Hospital of Xi'an Jiaotong University, Xi'an, 710061, Shaanxi, China; b School of Medicine, Yan'an University, Yan'an, 716000, Shaanxi, China; c Department of Geratology, The Ninth Hospital of Xi'an City, Xi'an, 710054, Shaanxi, China

**Keywords:** Cardiometabolic diseases, Coronary atherosclerotic heart disease, Machine learning algorithms, Risk model

## Abstract

**Background:**

Coronary atherosclerotic heart disease (CHD) is highly prevalent in Northwest China; however, effective preventive measures are limited. This study aimed to develop metabolic risk models tailored for the primary and secondary prevention of CHD in Northwest China.

**Methods:**

This hospital-based cross-sectional study included 744 patients who underwent coronary angiography. Data on demographic characteristics, comorbidities, and serum biochemical indices of the participants were collected. Three machine learning algorithms—recursive feature elimination, random forest, and least absolute shrinkage and selection operator—were employed to construct risk models. Model validation was performed using receiver operating characteristic and calibration curves, and the optimal cutoff values for significant risk factors were determined.

**Results:**

The predictive model for CHD onset included sex, overweight/obesity, and hemoglobin A1c (HbA1c) levels. For CHD progression to multiple coronary artery disease, the model included age, total cholesterol (TC), low-density lipoprotein cholesterol (LDL-C), and HbA1c levels. The model predicting an increased coronary Gensini score included sex, overweight/obesity, TC, LDL-C, high-density lipoprotein cholesterol, lipoprotein(a), and HbA1c levels. Notably, the optimal cutoff values for HbA1c and lipoprotein(a) for determining CHD progression were 6 % and 298 mg/L, respectively.

**Conclusions:**

Robust metabolic risk models were established, offering significant value for both the primary and secondary prevention of CHD in Northwest China. Weight loss, strict hyperglycemic control, and improvement in dyslipidemia may help prevent or delay the occurrence and progression of CHD in this region.

## 1 Introduction

Cardiometabolic diseases (CMDs), including type 2 diabetes (T2D) and cardiovascular disease (CVD), are the primary contributors to global mortality and morbidity [1]. Coronary atherosclerotic heart disease (CHD) is a major component of the global CVD burden [2] and serves as a crucial clinical phenotype of CMDs. In the United States, an estimated 20.1 million individuals have CHD, while 11.1 million Americans have chronic stable angina pectoris [3]. In China, the overall prevalence of CVD significantly increased by 14.7 % from 1990 to 2016, and CVD remains the leading cause of death [4].

The spatial patterns of the mortality and prevalence of CVD and its main subcategories, such as ischemic heart disease (IHD), vary significantly across China [4]. The gap in the relative burden of CVD between provinces widened from 1990 to 2016, with a more rapid decline observed in economically developed provinces [4]. From 1990 to 2015, 22 of 33 provinces experienced an increase in age-standardized mortality from IHD, with eight provinces experiencing an increase of over 30 % [5]. In particular, Qinghai Province in Northwest China saw a 54 % increase in IHD mortality and a 279 % increase in IHD deaths. However, 11 provinces showed a decreasing trend in IHD mortality, predominantly in economically developed regions [5]. The variations in CHD incidence and mortality rates, as well as the diverse patterns of change over short periods, suggest the potential for effective prevention strategies.

Exposure to cardiovascular risk factors, particularly metabolic disorders, has increased in China. Notably, the prevalence of hypertension increased from 7.7 % in 1980 to 27.5 % in 2018, whereas that of diabetes increased from 0.67 % in 1980 to 11.2 % in 2017 [6]. The 2015 China Adult Chronic Disease and Nutrition Surveillance Project indicated elevated cholesterol and triglyceride levels in Chinese adults compared with those in 2002 [7]. We hypothesized that the high incidence of CHD in Northwest China is strongly linked to specific metabolic risk factors. This study aimed to develop accurate metabolic risk models for CHD occurrence and progression in Northwest China and to determine the optimal cutoff values for key metabolic risk factors. Our findings will contribute to the development of effective CHD prevention strategies to reduce the burden of CMDs in this region.

## 2 Methods

### 2.1 Study design and participants

This cross-sectional study was conducted at a single center. We randomly selected 1000 individuals who presented with precardiac discomfort and underwent coronary angiography at the Department of Cardiovascular Medicine of the First Affiliated Hospital of Xi'an Jiaotong University between January 2022 and December 2022. This hospital is a major tertiary institution located in Northwest China and is renowned for its clinical practice, teaching, and scientific research activities.

The study included adult participants with complete clinical information who underwent coronary angiography. The exclusion criteria were as follows: (1) patients with a history of coronary artery bypass grafting or percutaneous coronary intervention; (2) patients with malignant tumors; and (3) patients who had previously been treated for thyroid disease with oral methimazole, propylthiouracil, amiodarone, thyroxine tablets, thyroid radioiodine-131 therapy, or thyroidectomy. Ultimately, 744 participants aged 23–90 years were included. Of these participants, 507 (68 %) were men, and 570 (77 %) were diagnosed with CHD. Among patients with CHD, 418 (73 %) had multiple coronary artery disease (CAD). Detailed information on the clinical and demographic characteristics of the study participants is presented in Table 1, Table 2.

**Table 1:** Clinical and demographical characteristics of the study participants.

Clinical characteristics	Control (N = 174)	CHD (N = 570)	*P-*value
Gensini score (IQR)	4 (7)	54 (62)	**<0.001**
Sex (%)			
male	94 (54.0)	413 (72.5)	**<0.001**
Female	80 (46.0)	157 (27.5)	
Age, years (IQR)	60 (15)	62 (16)	0.119
BMI, kg/m^2^ (IQR)	24.658 (3.8)	24.658 (2.1)	0.850
Overweight/obesity (%)			
yes	105 (60.3)	416 (73.0)	**<0.001**
no	69 (39.7)	154 (27.0)	
History of hypertension (%)			
yes	82 (47.1)	302 (53.0)	0.194
no	92 (52.9)	268 (47.0)	
SBP, mmHg	128.95 ± 19.03	134.23 ± 20.68	0.052
DBP, mmHg	79.95 ± 11.86	81.00 ± 12.81	0.358
History of diabetes (%)			
yes	22 (12.6)	122 (21.4)	**0.011**
no	152 (87.4)	448 (78.6)	
Tobacco use (%)			
yes	63 (36.2)	294 (51.6)	**<0.001**
no	111 (63.8)	276 (48.4)	
Alcohol use (%)			
yes	16 (9.2)	63 (11.1)	0.574
no	158 (90.8)	507 (88.9)	
Family history of CHD (%)			
yes	10 (5.7)	35 (6.1)	0.999
no	164 (94.3)	535 (93.9)	
Aspirin use (%)			
yes	43 (24.7)	144 (25.3)	0.921
no	131 (75.3)	426 (74.7)	
Statin use (%)			
yes	37 (21.3)	127 (22.2)	0.835
no	137 (78.7)	443 (77.7)	
eGFR<60 mL/min/1.73 m^2^ (%)			
yes	2 (1.1)	30 (5.3)	**0.018**
no	172 (98.9)	540 (94.7)	
eGFR, mL/min/1.73 m^2^ (IQR)	99.58 (14.32)	99.54 (19.29)	0.658
TC, mmol/L (IQR)	3.61 (1.02)	3.74 (1.24)	**0.049**
TG, mmol/L (IQR)	1.23 (0.83)	1.30 (0.87)	0.107
HDL-C, mmol/L (IQR)	0.98 (0.29)	0.93 (0.28)	**0.011**
LDL-C, mmol/L (IQR)	2.05 (0.96)	2.22 (1.09)	0.052
Lp(a), mg/L (IQR)	204.50 (239.00)	222.50 (238.25)	0.146
Fibrinogen, g/L (IQR)	2.78 (0.82)	3.10 (1.09)	**<0.001**
HbA1c, % (IQR)	5.80 (0.80)	6.00 (1.00)	**<0.001**
T4, ug/dL (IQR)	7.93 (2.98)	8.04 (2.36)	0.348
T3, ng/mL (IQR)	1.12 (0.32)	1.07 (0.31)	**0.030**
FT4, pmol/L (IQR)	16.05 (3.33)	15.97 (3.42)	0.889
FT3, pmol/L (IQR)	4.82 (1.41)	4.81 (1.14)	0.063
h-TSH, uIU/mL (IQR)	1.59 (1.38)	1.33 (1.58)	**0.041**

BMI, body mass index; CHD, coronary atherosclerotic heart disease; DBP, diastolic blood pressure; eGFR, estimated glomerular filtration rate; FT3, free triiodothyronine; FT4, free thyronine; HbA1c, hemoglobin A1c; HDL-C, high-density lipoprotein cholesterol; h-TSH, hypersensitive thyroid stimulating hormone; IQR, interquartile range; LDL-C, low-density lipoprotein cholesterol; Lp(a), lipoprotein(a); SBP, systolic blood pressure; TC, total cholesterol; TG, triglycerides; T3, triiodothyronine; T4, thyronine.

Significant *P*–values are indicated in bold.

**Table 2:** Clinical and demographical characteristics of patients with CHD.

Clinical characteristics	Number of coronary lesions		*P-*value	Gensini score		*P-*value
	Single-vessel CAD (N = 152)	Multiple CADs (N = 418)		≤53 (N = 281)	＞53 (N = 289)	
Gensini score (IQR)	23.50 (31.80)	71.50 (62.00)	**<0.001**			
Multiple CADs (%)						
yes				149 (53.02)	269 (93.08)	**<0.001**
no				132 (46.98)	20 (6.92)	
Sex (%)						
male	102 (67.11)	311 (74.40)	0.085	183 (65.12)	230 (79.58)	**<0.001**
female	50 (32.89)	107 (25.60)		98 (34.88)	59 (20.42)	
Age, years (IQR)	59（17）	63 (15)	**0.011**	61（15）	63 (16)	0.126
BMI, kg/m^2^ (IQR)	24.66（2.30）	24.66(2.00)	0.160	24.66（2.70）	24.66 (0.90)	0.958
Overweight/obesity (%)						
yes	104 (68.42)	312 (74.64)	0.139	195 (69.40)	221 (76.47)	0.057
no	48 (31.58)	106 (25.36)		86 (30.60)	68 (23.53)	
History of hypertension (%)						
yes	78 (51.32)	224 (53.59)	0.631	157 (55.87)	145 (50.17)	0.173
no	74 (48.68)	194 (46.41)		124 (44.13)	144 (49.83)	
SBP, mmHg	129.39 ± 20.22	133.44 ± 20.65	**0.037**	131.41 ± 19.54	133.29 ± 21.57	0.274
DBP, mmHg	81.43 ± 13.61	80.81 ± 12.94	0.615	80.30 ± 13.19	81.63 ± 13.02	0.225
History of diabetes (%)						
yes	24 (15.79)	98 (23.44)	**0.049**	49 (17.44)	73 (25.26)	**0.023**
no	128 (84.21)	320 (76.56)		232 (82.56)	216 (74.74)	
Tobacco use (%)						
yes	69 (45.39)	225 (53.83)	0.075	131 (46.62)	163 (56.40)	**0.019**
no	83 (54.61)	193 (46.17)		150 (53.38)	126 (43.60)	
Alcohol use (%)						
yes	19 (12.50)	44 (10.53)	0.224	34 (12.10)	29 (10.03)	0.432
no	133 (87.50)	374 (89.47)		247 (87.90)	260 (89.97)	
Family history of CHD (%)						
yes	9 (5.92)	26 (6.22)	0.895	20 (7.12)	15 (5.19)	0.338
no	143 (94.08)	392 (93.78)		261 (92.88)	274 (94.81)	
Aspirin use (%)						
yes	35 (23.03)	109 (26.08)	0.459	80 (28.47)	64 (22.15)	0.082
no	117 (76.97)	309 (73.92)		201 (71.53)	225 (77.85)	
Statin use (%)						
yes	28 (18.42)	99 (23.68)	0.182	65 (23.13)	62 (21.45)	0.630
no	124 (81.58)	319 (76.32)		216 (76.87)	227 (78.55)	
eGFR<60 mL/min/1.73 m^2^ (%)						
yes	5 (3.29)	25 (5.98)	0.203	11 (3.91)	19 (6.57)	0.155
no	147 (96.71)	393 (94.02)		270 (96.09)	270 (93.43)	
eGFR, mL/min/1.73 m^2^ (IQR)	103.16 (18.75)	98.70 (18.85)	**0.014**	101.00 (18.98)	98.27 (18.44)	0.062
TC, mmol/L (IQR)	3.64 (1.21)	3.77 (0.98)	0.198	3.72 (1.16)	3.77 (1.31)	0.250
TG, mmol/L (IQR)	1.33 (0.95)	1.29 (0.82)	0.888	1.29 (0.93)	1.32 (0.81)	0.641
HDL-C, mmol/L (IQR)	0.96 (0.27)	0.91 (0.29)	**0.043**	0.95 (0.32)	0.90 (0.20)	**0.014**
LDL-C, mmol/L (IQR)	2.10 (1.12)	2.23 (1.10)	0.188	2.14 (1.11)	2.25 (1.10)	0.097
Lp(a), mg/L (IQR)	197.00 (220.00)	229.00 (248.00)	**0.040**	209.00 (226.50)	231.00 (328.43)	**0.048**
Fibrinogen, g/L (IQR)	2.98 (0.99)	3.13 (1.38)	**0.006**	3.02 (0.97)	3.16 (1.18)	**0.006**
HbA1c, % (IQR)	5.90 (0.80)	6.10 (1.10)	**<0.001**	5.90 (0.80)	6.10 (1.20)	**<0.001**
T4, ug/dL (IQR)	8.04 (2.57)	8.04 (2.32)	0.955	8.04 (2.52)	8.04 (2.22)	0.665
T3, ng/mL (IQR)	1.04 (0.31)	1.07 (0.31)	0.585	1.09 (0.32)	1.04 (0.29)	0.284
FT4, pmol/L (IQR)	16.31 (3.30)	15.97 (3.49)	**0.044**	16.20 (3.42)	15.97 (3.40)	**0.002**
FT3, pmol/L (IQR)	4.82 (1.08)	4.79 (1.16)	0.193	4.81 (1.11)	4.72 (1.11)	**<0.001**
h-TSH, uIU/mL (IQR)	1.50 (1.51)	1.31 (1.59)	0.636	1.44 (1.53)	1.24 (1.64)	0.134

BMI, body mass index; CHD, coronary atherosclerotic heart disease; DBP, diastolic blood pressure; eGFR, estimated glomerular filtration rate; FT3, free triiodothyronine; FT4, free thyronine; HbA1c, hemoglobin A1c; HDL-C, high-density lipoprotein cholesterol; h-TSH, hypersensitive thyroid stimulating hormone; IQR, interquartile range; LDL-C, low-density lipoprotein cholesterol; Lp(a), lipoprotein(a); SBP, systolic blood pressure; TC, total cholesterol; TG, triglycerides; T3, triiodothyronine; T4, thyronine.

### 2.2 Observed variables

A total of 28 variables were included as candidate risk factors for CHD onset and progression based on previous literature and expert opinions. All 28 variables were classified into three categories, including metabolic factors. The demographic features of the study participants included sex, age, body mass index (BMI), overweight or obese status, smoking status, alcohol consumption status, and family history of CHD. The clinical variables included a history of hypertension, history of diabetes, systolic blood pressure (SBP), diastolic blood pressure, aspirin use, statin use, and presence of chronic kidney disease (estimated glomerular filtration rate [eGFR] <60 mL/min/1.73 m^2^). Additionally, we included blood biochemical indicators that may contribute to the risk assessment of CHD onset and progression. These biochemical indicators included hemoglobin A1c (HbA1c), eGFR, serum levels of total cholesterol (TC), triglycerides, low-density lipoprotein cholesterol (LDL-C), high-density lipoprotein cholesterol (HDL-C), lipoprotein(a) (Lp(a)), and plasma fibrinogen levels. Indicators of thyroid function, including thyroxine, triiodothyronine, free thyroxine, free triiodothyronine (FT3), and hypersensitive thyroid-stimulating hormone levels, were also included in this study. HbA1c levels were measured using high-performance liquid chromatography, and plasma fibrinogen levels were assessed using the class coagulation method. Serum triglyceride and TC levels were determined enzymatically using a colorimetric method, while HDL-C and LDL-C levels were measured directly. Serum Lp(a) levels were determined using an immunoturbidimetric method. Five thyroid function indices were measured using chemiluminescence assay. eGFR was calculated using the Chronic Kidney Disease Epidemiology Collaboration equation [8].

### 2.3 Outcome variables

All patients underwent coronary angiography via the right radial artery. CHD was diagnosed when the stenosis exceeded 50 % in any vessel of the main branches of the coronary artery, including the left anterior descending, left circumflex, and right coronary arteries. Stenosis of less than 50 % was considered non-CHD. Patients with CHD were further categorized into single- and multi-vessel CAD groups. Single-vessel CAD was defined as ≥50 % stenosis in one major coronary artery, while multi-vessel CAD was defined as ≥50 % stenosis in at least two vessels of the left anterior descending, left circumflex branches, and the right coronary artery. In addition, lesions with ≥50 % stenosis in the left main coronary artery were defined as multi-vessel CAD [9]. Additionally, patients with CHD were divided into two groups based on the median value of their coronary Gensini score: those with a low Gensini score (≤53) and those with a high Gensini score (>53). The Gensini score was calculated by summing the basic scores for each blood vessel multiplied by the corresponding scoring coefficient [10].

### 2.4 Statistical analysis

**Fig. 1:**
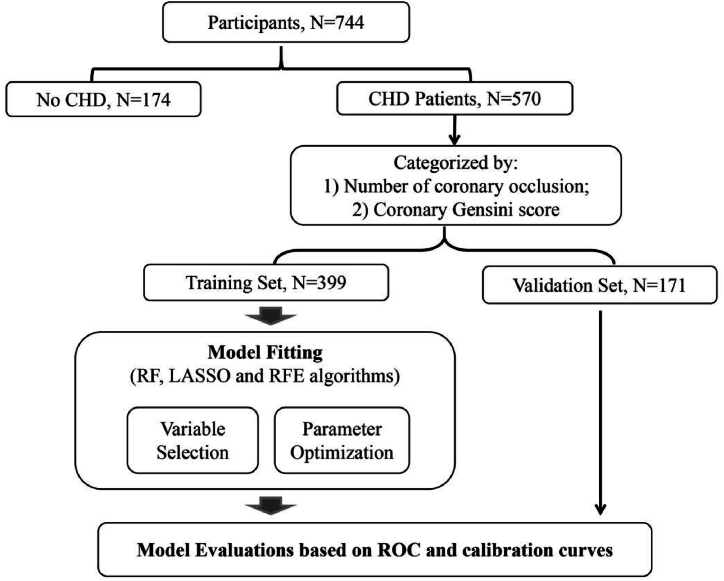
Study design and analysis framework. CHD, coronary atherosclerotic heart disease; LASSO, least absolute shrinkage and selection operator; RF, random forest; RFE, recursive feature elimination; ROC, receiver operating characteristic.

Machine learning models using random forest (RF), least absolute shrinkage and selection operator (LASSO), and recursive feature elimination (RFE) algorithms were fitted to evaluate the performance of multiple clinical and demographic variables as potential risk indicators of CHD occurrence and progression. A flowchart of the analysis is shown in Fig. 1.

Data from patients with CHD were randomly split into training and validation sets in a ratio of 7:3. Univariate logistic regression was used to screen risk factors in the training set, which were then optimized using RF, LASSO, and RFE. The support vector machine (SVM) method was adopted [11] for the training set to construct a diagnostic classifier for determining the occurrence and progression of CHD. Nomograms were constructed to predict the probability of CHD development. The model performance was evaluated using receiver operating characteristic (ROC) curves, calibration curves, area under the curve (AUC), C-index, and Youden's index for optimal cutoff values.

The Kolmogorov–Smirnov test was used to assess normality. Continuous variables are presented as mean ± standard deviation (SD) and compared using t-tests. Non-normal variables are presented as the median and interquartile range and compared using the Mann–Whitney *U* test. Categorical variables are expressed as percentages and compared using the chi-square test. Statistical significance was set at P < 0.05. Analyses were conducted using the R software (v3.6.1).

## 3 Results

### 3.1 Participant characteristics

In total, 744 participants were recruited, including 570 and 174 with and without CHD, respectively. Table 1 summarizes the patients’ clinical and demographic characteristics. Among the 28 features studied, 12 were significantly different between the two groups based on the CHD status. For those with CHD, 10 features were significantly different from those with and without multiple CADs. Similarly, 10 features were significantly different among patients with CHD based on their Gensini scores (Table 2).

### 3.2 Screening for potential risk factors

In the training set, univariate logistic regression was used to screen for risk factors significantly associated with each outcome. We screened five risk factors significantly associated with CHD onset: sex, overweight/obesity, tobacco use, HDL-C, and HbA1c; five risk factors significantly associated with multiple CADs: age, SBP, TC, LDL-C, and HbA1c levels; and eight risk factors significantly associated with increased Gensini scores: sex, overweight/obesity, TC, HDL-C, LDL-C, Lp(a), HbA1c, and FT3 levels. The odds ratios of the selected features for the three outcomes are presented in Supplemental Table S1.

### 3.3 Identification of optimal risk factors

**Fig. 2:**
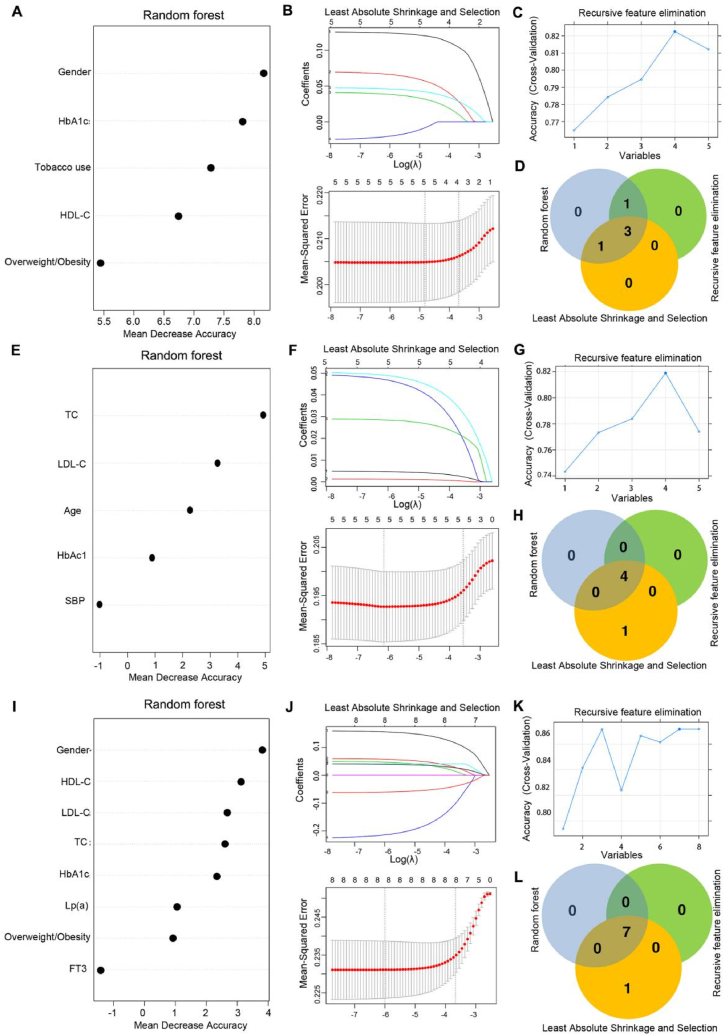
Optimal screening of risk factors for CHD onset and progression. A Parameter diagram of risk factors for CHD onset screened using the random forest algorithm. **B** Parameter graphs of risk factors for CHD onset screened using the least absolute shrinkage and selection operator algorithm. **C** Parameter diagram of risk factors for CHD onset screened using the recursive feature elimination algorithm. **D** Comparative Venn diagram of risk factors for CHD onset screened using the three algorithms. **E** Parameter diagram of risk factors for multiple CADs screened using the random forest algorithm. **F** Parameter graphs of risk factors for multiple CADs screened using the least absolute shrinkage and selection operator algorithm. **G** Parameter diagram of risk factors for multiple CADs screened using the recursive feature elimination algorithm. **H** Comparative Venn diagram of risk factors for multiple CADs screened using the three algorithms. **I** Parameter diagram of risk factors for a high Gensini score screened using the random forest algorithm. **J** Parameter graphs of risk factors for a high Gensini score screened using the least absolute shrinkage and selection operator algorithm. **K** Parameter diagram of risk factors for a high Gensini score screened using the recursive feature elimination algorithm. **L** Comparative Venn diagram of risk factors for high Gensini scores screened using the three algorithms. CAD, coronary artery disease; CHD, coronary atherosclerotic heart disease; FT3, free triiodothyronine; HbA1c, hemoglobin A1c; HDL-C, high-density lipoprotein cholesterol; LDL-C, low-density lipoprotein cholesterol; Lp(a), lipoprotein(a); SBP, systolic blood pressure; TC, total cholesterol.

Based on the univariate logistic regression analysis, we utilized the RF, LASSO, and RFE algorithms to refine risk factor screening for the three outcomes: CHD, multiple CADs, and Gensini score. For CHD, these algorithms identified overlapping clinical factors such as sex, overweight/obesity, and HbA1c levels. Similarly, for multiple CADs, the optimized factors included age, TC, LDL-C, and HbA1c levels. Seven clinical factors were identified for the Gensini score: sex, overweight/obesity, TC, HDL-C, LDL-C, Lp(a), and HbA1c levels. The algorithm parameters are presented in Fig. 2.

### 3.4 Development and validation of SVM diagnostic classification models

**Fig. 3:**
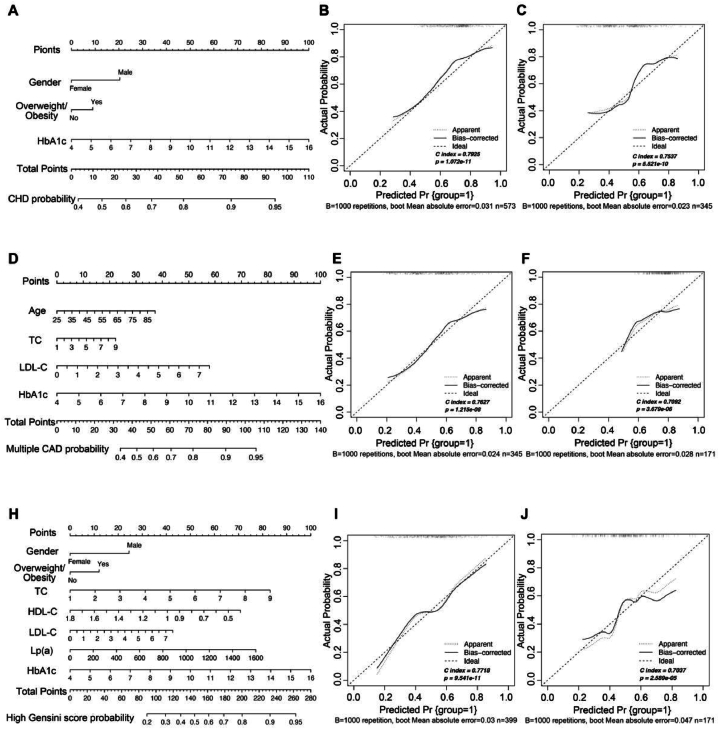
Nomograms and calibration plots of the predictive models. **A** Nomogram built with the training set for CHD onset. **B** Calibration curve of the training set for CHD onset. **C** Calibration curve of the validation set for CHD onset. **D** Nomogram built with the training set for multiple CADs. **E** Calibration curve of the training set for multiple CADs. **F** Calibration curve of the validation set for multiple CADs. **G** Nomogram built with the training set for a high Gensini score. **H** Calibration curve of the training set for a high Gensini score. **I** Calibration curve of the validation set for a high Gensini score. CAD, coronary artery disease; CHD, coronary atherosclerotic heart disease; HbA1c, hemoglobin A1c; HDL-C, high-density lipoprotein cholesterol; LDL-C, low-density lipoprotein cholesterol; Lp(a), lipoprotein(a); TC, total cholesterol.

Furthermore, we established three SVM diagnostic classification models for predicting CHD risk, multiple CADs, and increased CAD Gensini scores and plotted their respective nomograms in the training set (Fig. 3A, D, 3G). Calibration plots were drawn, and the C-index was calculated to evaluate the predictive ability of the three models. For the CHD occurrence model, the C-index was 0.793 in the training set (Figs. 3B) and 0.754 in the validation set (Fig. 3C). For the multiple CAD model, the C-index was 0.763 in the training set (Figs. 3E) and 0.709 in the validation set (Fig. 3F). For the model with an increased Gensini score, the C-index was 0.772 in the training set (Figs. 3H) and 0.704 in the validation set (Fig. 3I).

### 3.5 Model performance

**Fig. 4:**
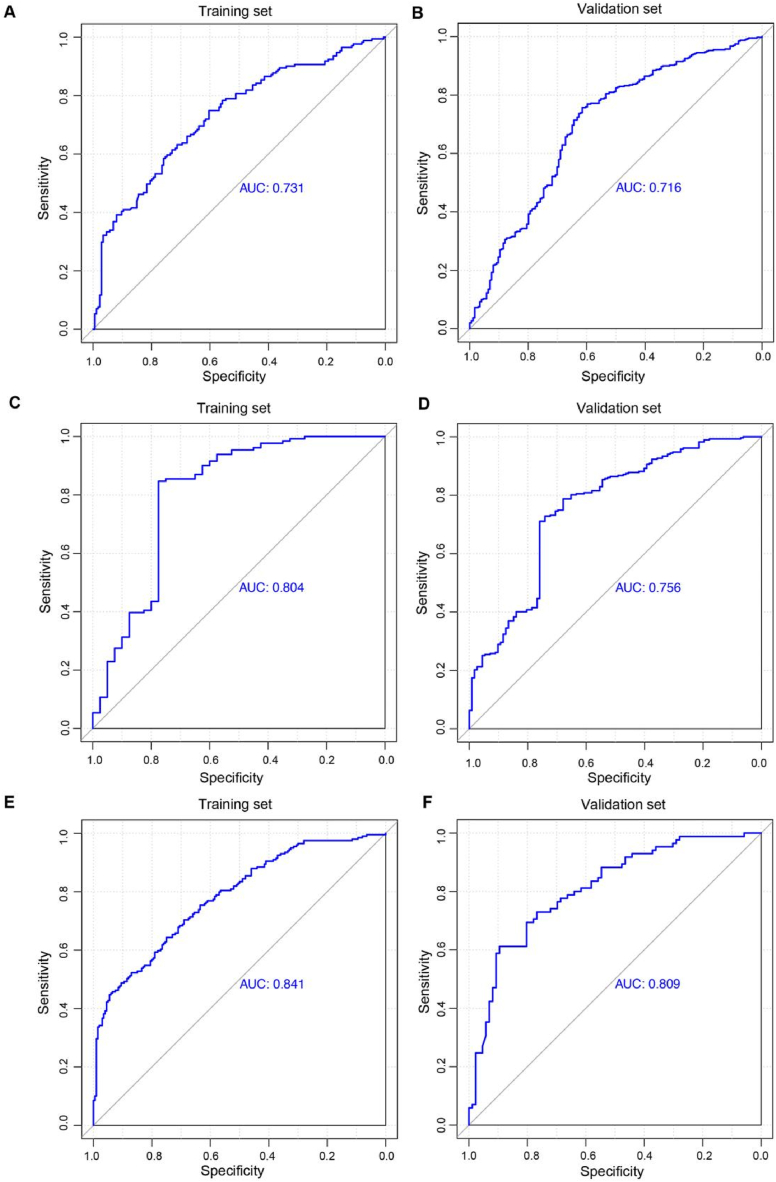
ROC curves for evaluating CHD occurrence and progression using the SVM model. **A** ROC curve of multiple clinical features predicting CHD risk in the training set. **B** ROC curve of multiple clinical features predicting CHD risk in the validation set. **C** ROC curve of multiple clinical features predicting multiple CADs in the training set. **D** ROC curve of multiple clinical features predicting multiple CADs in the validation set. **E** ROC curve of multiple clinical features predicting a high Gensini score in the training set. **F** ROC curve of multiple clinical features predicting a high Gensini score in the validation set. AUC, area under the curve; CAD, coronary artery disease; CHD, coronary atherosclerotic heart disease; HbA1c, hemoglobin A1c; HDL-C, high-density lipoprotein cholesterol; LDL-C, low-density lipoprotein cholesterol; Lp(a), lipoprotein(a); ROC, receiver operating characteristic; SVM, support vector machine; TC, total cholesterol.

The ROC curves were constructed to evaluate the effectiveness of each model on the training and validation sets. For the outcome classified according to CHD onset, the ability of the model established by combining sex, overweight/obesity, and HbA1c levels to identify CHD was significantly greater than that of any single factor. The AUC values for this model were 0.731 and 0.716 for the training (Fig. 4A) and validation sets (Fig. 4B), respectively. For the outcome classified by multiple CADs, the ability of the model established using age, TC, LDL-C, and HbA1c levels to identify CHD progression was significantly greater than that of any single factor, with AUC values of 0.804 in the training set (Figs. 4C) and 0.756 in the validation set (Fig. 4D). For the outcome classified by the Gensini score, the ability of the model established using sex, overweight/obesity, TC, HDL-C, LDL-C, Lp(a), and HbA1c levels to identify CHD progression was significantly greater than that of any single factor, with AUC values of 0.841 in the training set (Figs. 4E) and 0.809 in the validation set (Fig. 4F). The AUC values of each ROC curve and the corresponding 95 % confidence intervals are shown in Supplemental Table S2.

### 3.6 Optimal cutoff values for the single-indicator model with HbA1c or Lp(a) levels

**Fig. 5:**
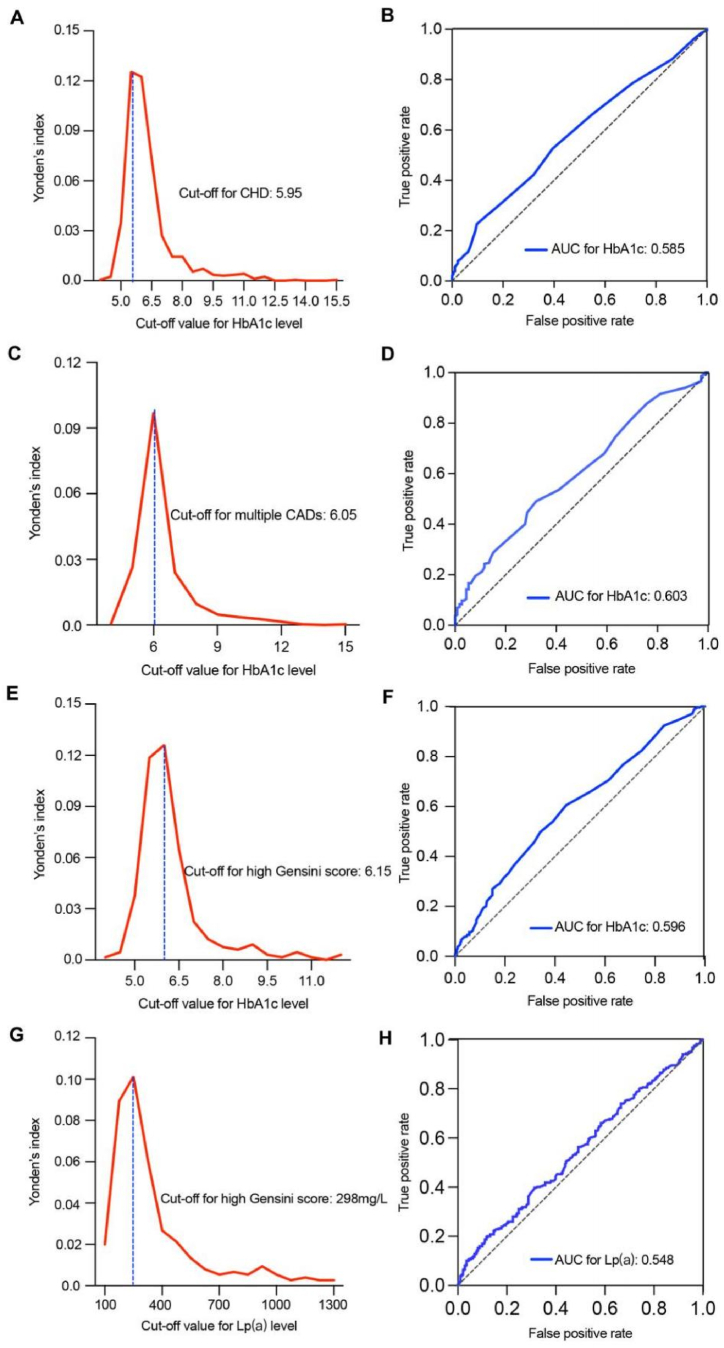
Cutoff values and model performance of the single important feature. **A** Youden's index and the cutoff value for CHD onset using the HbA1c level as a single feature. **B** ROC curve and AUC value for CHD onset using the HbA1c level as a single feature. **C** Youden's index and the cutoff value for multiple CADs using the HbA1c level as a single feature. **D** ROC curve and AUC value for multiple CADs using the HbA1c level as a single feature. **E** Youden's index and the cutoff value for a high Gensini score in CHD using the HbA1c level as a single feature. **F** ROC curve and AUC value for high Gensini score in CHD using the HbA1c level as a single feature. **G** Youden's index and the cutoff value for a high Gensini score in CHD using the Lp(a) level as a single feature. **H** ROC curve and AUC value for a high Gensini score in CHD using the Lp(a) level as a single feature. AUC, area under the curve; CAD, coronary artery disease; CHD, coronary atherosclerotic heart disease; HbA1c, hemoglobin A1c; Lp(a), lipoprotein(a); ROC, receiver operating characteristic.

The performance of the single-indicator models, including HbA1c and Lp(a) levels, was examined to predict the occurrence and progression of CHD. The optimal cutoff values for HbA1c were 5.95 %, 6.05 %, and 6.15 % for CHD risk, multiple CADs, and increased CAD Gensini scores, respectively (Fig. 5A, C, 5E), with AUC values of 0.585, 0.603, and 0.596, respectively (Fig. 5B, D, 5F). For Lp(a), the optimal cutoff value was 298 mg/L (Fig. 5G), with an AUC of 0.548 (Fig. 5H). The sensitivity and specificity of these models are summarized in Supplemental Table S3.

## 4 Discussion

To the best of our knowledge, our study is the first to use three machine learning algorithms to investigate the metabolic risk factors influencing the initiation and advancement of CHD in Northwest China. This study presents four major findings. First, we developed a metabolic risk model for CHD onset, with predictors including sex, overweight/obesity, and HbA1c levels. Second, we established a metabolic risk model to evaluate CHD progression to multiple CADs, with predictors including age, TC, LDL-C, and HbA1c levels. Third, we developed a metabolic risk model to assess CHD progression to an increased CAD Gensini score, with predictors including sex, overweight/obesity, TC, LDL-C, HDL-C, Lp(a), and HbA1c levels. Fourth, the optimal cutoff values of HbA1c and Lp(a) levels for determining CHD progression were approximately 6.0 % and 298 mg/L, respectively.

Hyperglycemia is a significant metabolic risk factor for atherosclerotic cardiovascular disease (ASCVD) [12]. The growing prevalence of diabetes has led to an increase in CMDs and related deaths in China, particularly in the Northwest region [6]. This study found that higher HbA1c levels increased the risk of CHD occurrence and progression, and the HbA1c level was the most significant factor in the assessment model for CHD, emphasizing the importance of managing hyperglycemia in developing prevention strategies for CHD in this region. Furthermore, this study found that HbA1c levels of >6 % increased the risk of CHD, highlighting the need for routine HbA1c screening, early detection of prediabetes/diabetes, and maintenance of HbA1c levels of <6 % to reduce the risk of CHD. Multiple clinical trials have shown that lifestyle interventions coupled with reduced-calorie meal plans effectively prevent or delay T2D [[13], [14], [15]] and improve cardiometabolic markers [16]. The 2024 American Diabetes Association guideline recommends lifestyle changes, including a healthy diet and ≥150 min/week of moderate-intensity physical activity, to reduce weight by at least 7 % for adults with overweight/obesity who are at a high risk of T2D [12]. Notably, achieving lower HbA1c levels safely without hypoglycemia is also beneficial [17]. However, cardiovascular event rates remain high in patients with T2D despite good glycemic control [18]; thus, glucose-lowering medications with proven cardiovascular benefits are recommended for patients with CHD and T2D [19,20].

This study underscores the importance of addressing overweight and obesity in Northwest China to reduce the risk of CHD development and progression. Compared to normal-weight individuals, patients with obesity experience chronic coronary disease (CCD) events at an earlier age, live with CCD for a greater proportion of their lifetime, and have a shorter average life span [21]. Excess adiposity accelerates atherosclerosis and promotes adverse changes in cardiac function by exerting deleterious effects on the myocardium, vasculature, and obesity-related comorbidities, such as hypertension, dyslipidemia, and T2D [22,23]. Compared with those in the eastern coastal areas, people in Northwest China consume more carbohydrates and fewer vegetables and fruits and exercise less outdoors owing to a colder winter weather. These habits may lead to a high incidence of being overweight or obese in this region, further increasing the risk of CHD. The latest guidelines recommend that, in patients with CCD, the assessment of BMI with or without waist circumference is recommended during routine clinical follow-up [20]. For patients who require pharmacological therapy for further weight reduction, drug therapies can be effective alongside counseling regarding diet and physical activity [24]. In patients with CCD and severe obesity who have not met weight loss goals with lifestyle and pharmacological intervention and have acceptable surgical risk, referral for a bariatric procedure is reasonable for weight loss and cardiovascular risk reduction [25,26]. Therefore, altering unhealthy dietary and behavioral patterns in Northwest China and adopting beneficial lifestyles and dietary patterns recommended by the guidelines can minimize the risk of being overweight or obese, thus reducing the burden of CMDs.

This study highlights the significant role of elevated LDL-C, TC, and Lp(a) levels in promoting CAD progression in Northwest China, suggesting that inadequate lipid management during the secondary prevention of CHD could be a primary factor contributing to the high burden of CHD in this region. Importantly, elevated LDL-C levels are major contributors to ASCVD [27]. The cornerstone of managing serum cholesterol levels is promoting a healthy lifestyle throughout one's lifetime [28]. Even individuals with a genetic predisposition to CHD can reduce their risk by up to 50 % through lifestyle modifications [29]. Strategies such as maintaining normal weight and blood sugar levels, reducing the intake of simple sugars and refined carbohydrates, and increasing physical activity can improve lipid profiles and provide additional health benefits [30]. Currently, the medications available for LDL-C reduction include statins, ezetimib, proprotein convertase subtilisin/kexin type 9 inhibitors, and inclisiran [31].

Furthermore, this study is the first to demonstrate that serum Lp(a) levels >298 mg/L contribute to increased CAD Gensini scores in patients with CHD. High Lp(a) levels are known to cause ASCVD and cardiovascular and all-cause mortality in both men and women and in ethnically diverse populations [32]. The mechanisms by which Lp(a) increases the risk of ASCVD are diverse. First, Lp(a) particles contain apolipoprotein B, similar to other apolipoprotein B-containing particles such as LDL, conferring atherogenic properties. Second, Lp(a) serves as a significant carrier of oxidized phospholipids, linked to damage and capable of triggering pro-inflammatory responses. Finally, it is hypothesized that apolipoprotein(a) partially and selectively binds to endothelial extracellular matrix proteins, leading to its retention within the arterial wall [33]. As Lp(a) levels are genetically determined, lifestyle interventions do not affect Lp(a)-mediated ASCVD risk. Genetically determined Lp(a) levels are not influenced by lifestyle interventions, and current lipid-lowering therapies have a limited clinical impact on Lp(a) levels. However, there are multiple Lp(a)-directed therapies in clinical development that target *LPA* mRNA, such as pelacarsen [34], olpasiran [35], and SLN360 [36], which have demonstrated the ability to reduce plasma Lp(a) levels by up to 90 %. Although the exact reduction required to achieve clinically meaningful benefits remains uncertain, our findings indicate that maintaining serum Lp(a) levels below 298 mg/L may potentially delay the progression of CHD.

Our study has several strengths. First, our model, derived from various observational variables of patients in our region, exhibited distinct racial, regional, and temporal characteristics, enhancing its applicability to the local population compared to that of previous methods. Second, metabolic factors have been recognized as contributors to the development and progression of ASCVD [[37], [38], [39]]. Hence, our model incorporates traditional risk factors along with novel variables such as thyroid function, HbA1c, and Lp(a). Third, we utilized advanced machine learning algorithms to construct a multifactorial integrated prediction model for CHD, leveraging various information sources to enhance prediction accuracy. Finally, our research provides optimal cutoff values for HbA1c for predicting the onset and progression of CHD, thereby supporting glycemic control goals for the prevention and management of CMDs.

This study has some limitations. First, cross-sectional data were utilized, preventing the establishment of a temporal relationship between the risk factors and CHD onset and progression. Further prospective studies are required to validate this association. Second, the lack of data on newly identified variables, including predisposition genes, may have hindered our ability to identify additional risk factors.

## 5 Conclusions

In summary, we developed effective metabolic risk models to evaluate the occurrence and progression of CHD in Northwest China. These models provide a crucial foundation for enhancing preventive strategies against CHD by identifying and managing the combined metabolic risk factors. Prioritizing strict interventions for hyperglycemia is expected to mitigate the risk of CHD occurrence and progression in this region.

## Ethics statement

This study was reviewed and approved by the Ethics Committee of the First Affiliated Hospital of Xi'an Jiaotong University (approval number: XJTU1AF2019LSK-064; dated June 25, 2019). All participants provided written informed consent, and their data were approved for publication.

## Funding

This work was supported by Grants from the 10.13039/501100001809National Natural Science Foundation of China [81970329], Clinical Research Award of the First Affiliated Hospital of Xi'an Jiaotong University, China [XJTU1AF-CRF-2019-030], Shaanxi Province Science Technology of China [2019KW-079], Xi'an Innovation Capability Support Program Medical Research Project [23YXYJ0096], and Ninth Hospital of Xi'an City General Research [2022yb01, 2022yb20].

## Data availability statement

Data will be made available upon reasonable request.

## CRediT authorship contribution statement

**Qingfeng Wu:** Writing – original draft, Formal analysis. **Huiyi Wei:** Methodology, Formal analysis. **Cong Lu:** Formal analysis. **Xiaoxian Chi:** Investigation. **Rongfang Li:** Funding acquisition. **Qingbin Zhao:** Writing – review & editing, Supervision, Funding acquisition, Conceptualization.

## Declaration of competing interest

The authors declare that they have no known competing financial interests or personal relationships that could have appeared to influence the work reported in this paper.
