# Supplementary material for: Establishment of precise prevention strategies for the occurrence and progression of coronary atherosclerotic heart disease using machine learning
Source: Heliyon. 2024 Aug 3;10(15):e35797. doi: 10.1016/j.heliyon.2024.e35797 (PMC11337032; doi:10.1016/j.heliyon.2024.e35797)
Supplement: Multimedia component 1 [file mmc1.docx]

**Supplementary Tables**

**Table S1** Odds ratios (OR) for different features measured using univariate logistic regression in the training set

|  | **CHD vs. Non-CHD** |  | **Multiple vs. Single CAD** |  | **High vs. Low Gensini score** |  |
| --- | --- | --- | --- | --- | --- | --- |
| **Clinical characteristics** | **OR (95% CI)** | ***P-*value** | **OR (95% CI)** | ***P-*value** | **OR (95% CI)** | ***P-*value** |
| Sex (male/female) | 2.150 (1.490–3.120) | **<0.001** | 1.220 (0.760–1.960) | 0.418 | 2.060 (1.320–3.220) | **0.002** |
| Age (years) | 1.010 (0.990–1.020) | 0.345 | 1.020 (1.000–1.040) | **0.019** | 1.010 (0.990–1.030) | 0.214 |
| BMI (kg/m^2^) | 0.980 (0.920–1.050) | 0.539 | 1.030 (0.940–1.120) | 0.497 | 0.980 (0.910–1.060) | 0.627 |
| Overweight/obesity (yes/no) | 1.620 (1.120–2.360) | **0.011** | 1.570 (0.980–2.510) | 0.059 | 1.670 (1.070–2.580) | **0.023** |
| SBP (mmHg) | 1.000 (1.000–1.010) | 0.305 | 1.010 (1.000–1.020) | **0.035** | 1.010 (1.000–1.020) | 0.228 |
| DBP (mmHg) | 1.000 (0.990–1.020) | 0.612 | 1.000 (0.980–1.020) | 0.944 | 1.010 (0.990–1.020) | 0.222 |
| History of hypertension (yes/no) | 1.300 (0.910–1.850) | 0.152 | 1.170 (0.750–1.800) | 0.493 | 0.930 (0.630–1.380) | 0.728 |
| History of diabetes (yes/no) | 1.570 (0.940–2.630) | 0.084 | 1.520 (0.830–2.780) | 0.173 | 1.400 (0.840–2.340) | 0.191 |
| Tobacco use (yes/no) | 1.820 (1.270–2.630) | **0.001** | 1.280 (0.830–1.980) | 0.267 | 1.370 (0.920–2.020) | 0.121 |
| Alcohol use (yes/no) | 1.010 (0.550–1.870) | 0.976 | 0.800 (0.380–1.640) | 0.536 | 0.660 (0.330–1.310) | 0.236 |
| Family history of CHD (yes/no) | 1.380 (0.660–2.880) | 0.390 | 1.130 (0.490–2.610) | 0.77 | 0.710 (0.340–1.480) | 0.359 |
| Aspirin use (yes/no) | 1.060 (0.700–1.600) | 0.781 | 1.130 (0.680–1.880) | 0.626 | 0.840 (0.530–1.310) | 0.441 |
| Statin use (yes/no) | 1.140 (0.740–1.760) | 0.548 | 1.270 (0.750–2.160) | 0.375 | 0.950 (0.600–1.510) | 0.835 |
| TC (mmol/L) | 1.180 (0.960–1.450) | 0.125 | 1.440 (1.110–1.880) | **0.006** | 1.310 (1.050–1.640) | **0.017** |
| TG (mmol/L) | 1.030 (0.900–1.190) | 0.670 | 1.160 (0.900–1.490) | 0.24 | 1.120 (0.930–1.350) | 0.222 |
| HDL-C (mmol/L) | 0.400 (0.190–0.860) | **0.019** | 0.590 (0.230–1.530) | 0.282 | 0.340 (0.140–0.830) | **0.018** |
| LDL-C (mmol/L) | 1.210 (0.940–1.540) | 0.133 | 1.500 (1.100–2.040) | **0.01** | 1.430 (1.100–1.860) | **0.008** |
| Lp(a) (mg/L) | 1.000 (0.996–1.020) | 0.129 | 1.000 (0.998–1.001) | 0.177 | 1.010 (1.001–1.030) | **0.008** |
| Fibrinogen (g/L) | 1.140 (0.970–1.340) | 0.118 | 1.180 (0.960–1.450) | 0.106 | 1.150 (0.980–1.370) | 0.094 |
| eGFR<60 (yes/no) | 4.300 (0.990–8.660) | 0.051 | 1.490 (0.480–4.590) | 0.488 | 1.120 (0.450–2.830) | 0.806 |
| eGFR (mL/min/1.73m^2^ ) | 0.990 (0.990–1.000) | 0.231 | 0.990 (0.980–1.010) | 0.304 | 0.990 (0.980–1.000) | 0.131 |
| HbA1c (%) | 1.350 (1.110–1.640) | **0.002** | 1.490 (1.160–1.900) | **0.002** | 1.240 (1.060–1.460) | **0.009** |
| T4 (ug/dL) | 0.990 (0.910–1.070) | 0.812 | 1.000 (0.910–1.110) | 0.93 | 0.980 (0.890–1.070) | 0.586 |
| T3 (ng/mL) | 0.970 (0.890–1.080) | 0.762 | 0.950 (0.820–1.090) | 0.434 | 0.990 (0.860–1.140) | 0.89 |
| FT4 (pmol/L) | 1.000 (0.950–1.060) | 0.927 | 0.970 (0.910–1.040) | 0.364 | 0.950 (0.890–1.020) | 0.153 |
| FT3 (pmol/L) | 0.920 (0.770–1.090) | 0.328 | 0.890 (0.710–1.110) | 0.3 | 0.750 (0.610–0.940) | **0.01** |
| h-TSH (uIU/mL) | 0.990 (0.970–1.020) | 0.537 | 1.030 (0.960–1.100) | 0.423 | 0.990 (0.960–1.030) | 0.696 |

BMI, body mass index; CAD, coronary artery disease; CHD, coronary atherosclerotic heart disease; CI, confidence interval; DBP, diastolic blood pressure; eGFR, estimated glomerular filtration rate; FT3, free triiodothyronine; FT4, free thyroxine; HbA1c, hemoglobin A1c; HDL-C, high-density lipoprotein cholesterol; h-TSH, hypersensitive thyroid stimulating hormone; LDL-C, low-density lipoprotein cholesterol; Lp(a), lipoprotein(a); OR, odds ratio; SBP, systolic blood pressure; TC, total cholesterol; TG, triglyceride; T3, triiodothyronine; T4, thyroxine.

Significant *P*-values are indicated in bold.

**Table S2** Area under the curve and 95% confidence interval values for different models

| **Model** | **AUC (95% CI)**  **(Training set)** | **AUC (95% CI)**  **(Validation set)** |
| --- | --- | --- |
| Risk of CHD |  |  |
| Sex | 0.611 (0.579–0.643) | 0.608 (0.576–0.640) |
| Overweight/obesity | 0.624 (0.592–0.656) | 0.603 (0.571–0.635) |
| HbA1c | 0.672 (0.640–0.704) | 0.659 (0.627–0.691) |
| Combination | 0.731 (0.699–0.763) | 0.716 (0.684–0.748) |
| CHD patients classified by the number of coronary occlusions |  |  |
| Age | 0.656 (0.624–0.688) | 0.619 (0.587–0.651) |
| TC | 0.617 (0.585–0.649) | 0.656 (0.624–0.688) |
| LDL-C | 0.62 (0.588–0.652) | 0.59 (0.558–0.622) |
| HbA1c | 0.686 (0.654–0.718) | 0.578 (0.546–0.610) |
| Combination | 0.804 (0.772–0.836) | 0.756 (0.724–0.788) |
| CHD patients classified by Gensini score |  |  |
| Sex | 0.572 (0.540–0.604) | 0.569 (0.537–0.601) |
| Overweight/obesity | 0.587 (0.555–0.619) | 0.578 (0.546–0.610) |
| TC | 0.607 (0.575–0.639) | 0.636 (0.604–0.668) |
| HDL-C | 0.655 (0.623–0.687) | 0.574 (0.542–0.606) |
| LDL-C | 0.634 (0.602–0.666) | 0.595 (0.563–0.627) |
| Lp(a) | 0.688 (0.656–0.720) | 0.608 (0.576–0.640) |
| HbA1c | 0.643 (0.611–0.675) | 0.607 (0.575–0.639) |
| Combination | 0.841 (0.809–0.873) | 0.809 (0.777–0.841) |

AUC, area under the curve; CHD, coronary atherosclerotic heart disease; CI, confidence interval; HbA1c, hemoglobin A1c; HDL-C, high-density lipoprotein cholesterol; LDL-C, low-density lipoprotein cholesterol; Lp(a), lipoprotein(a); TC, total cholesterol.

**Table S3** Sensitivity and specificity of single-marker models using Youden's index cutoff

| **Model** | **Sensitivity (95% CI)** | **Specificity (95% CI)** |
| --- | --- | --- |
| HbA1c |  |  |
| CHD | 0.527 (0.485–0.567) | 0.606 (0.532–0.675) |
| Multiple CAD | 0.491 (0.434–0.549) | 0.679 (0.587–0.758) |
| High Gensini score | 0.477 (0.409–0.547) | 0.675 (0.607–0.736) |
| Lp(a) |  |  |
| High Gensini score | 0.398 (0.343–0.455) | 0.687 (0.630–0.738) |

CAD, coronary artery disease; CHD, coronary atherosclerotic heart disease; CI, confidence interval; HbA1c, hemoglobin A1c; Lp(a), lipoprotein(a).
